# Spurious transcription factor binding: Non-functional or genetically redundant?

**DOI:** 10.1002/bies.201400036

**Published:** 2014-05-30

**Authors:** Mikhail Spivakov

**Affiliations:** 1 Babraham InstituteCambridge, UK

**Keywords:** functional genomics, genetic redundancy, regulatory variation, transcription factors, transcriptional regulation

## Abstract

Transcription factor binding sites (TFBSs) on the DNA are generally accepted as the key nodes of gene control. However, the multitudes of TFBSs identified in genome-wide studies, some of them seemingly unconstrained in evolution, have prompted the view that in many cases TF binding may serve no biological function. Yet, insights from transcriptional biochemistry, population genetics and functional genomics suggest that rather than segregating into ‘functional’ or ‘non-functional’, TFBS inputs to their target genes may be generally cumulative, with varying degrees of potency and redundancy. As TFBS redundancy can be diminished by mutations and environmental stress, some of the apparently ‘spurious’ sites may turn out to be important for maintaining adequate transcriptional regulation under these conditions. This has significant implications for interpreting the phenotypic effects of TFBS mutations, particularly in the context of genome-wide association studies for complex traits.

## Introduction

Sequence-specific transcription factors (TFs) are key regulators of gene expression in time and space, and their binding sites on the DNA (transcription factor binding sites [TFBSs]) are classically thought to represent highly specific functional elements. As expected from this understanding, many TFBSs are evolutionary conserved and their perturbation in a number of well-studied cases leads to strong changes in transcriptional activity in reporter assays conducted in vitro and in transgenic models 1,2. Genome-wide analyses of transcription factor binding by chromatin immunoprecipitation (ChIP) have however challenged this paradigm, revealing that TFs bind thousands of genomic locations in the vicinity of both active and inactive regions 3,4. A number of weakly bound sites detected this way failed to drive transgenic reporter expression 5. Evolutionary analyses of TFBS consensus sequences at in vivo-bound sites have delivered an additional surprise, demonstrating that in some cases they are no more evolutionary conserved than the flanking sequence, even at transcriptionally active regions 6–9. Finally, some regions in the genome (termed high occupancy transcription or HOT regions) have been found to co-recruit dozens of TFs without a strong sequence consensus for many of them 10–13. These findings have brought into question the concept of a uniformly ‘functional’ transcription factor recruitment that occurs only when and where necessary, leading to the notion of ‘non-functional’ or ‘spurious’ binding 3,14,15.

It can be argued however that rather than segregating TF-binding events into ‘functional’ and ‘non-functional’, it may be more appropriate to view them on a continuum defined by the potency of their regulatory outputs and the extent to which these outputs are redundant. As will be discussed in this essay, this view is in agreement with the current understanding of the mechanics of transcriptional activation as well as with evidence from genomics and population genetics. In particular, it offers an explanation as to why TFBSs with very similar properties may appear to be either functional or non-functional in different contexts. It also cautions that some apparently ‘useless’ TFBSs may become instrumental in conditions that affect the degree of genetic redundancy, such as mutations and environmental stress.

This essay will focus on transcriptional activation as one of the key functional outcomes of TF binding. It goes without saying that TFs also play other fundamental roles, such as facilitating gene repression, setting chromatin boundaries and maintaining ‘primed’ transcriptional states 16–18. Although not considered here, it is likely that a similar reasoning can be applied for interpreting multiple TF binding events in these contexts.

## The many recipes for transcriptional activation

In the classic scenario, transcriptional activation by TFs starts by their binding to their respective recognition sites on the DNA. The majority of sequence-specific TFs possess no enzymatic activity of their own and their main function likely lies in recruiting core co-factors, including, among others, histone modifying enzymes, chromatin remodelling factors and the mediator complex. These co-factors, in turn, ‘open’ and remodel the chromatin and promote the recruitment of RNA polymerase II along with the general TFs (TFIIA to TFIIH) that together form the pre-initiation complex 19,20. The polymerase then either proceeds to active elongation or remains in the paused (serine-5-phosphorylated) form; this transition is also regulated by sequence-specific TFs and the chromatin structure 21. Additionally, if transcriptional activation is initiated at remote regulatory modules (enhancers), as it often is, particularly at developmental genes, another necessary step is the establishment of DNA looping interactions between these remote regions and their target promoters 22. Mechanisms underlying the formation of looping interactions are not fully understood, but are known to involve sequence-specific TFs and structural proteins, such as CTCF and cohesin 23. Evidence from in vivo imaging also suggests that looping interactions may be stabilised at specific nuclear foci, termed ‘transcription factories’ that are enriched for proteins involved in transcriptional initiation 24,25.

**Figure 1:**
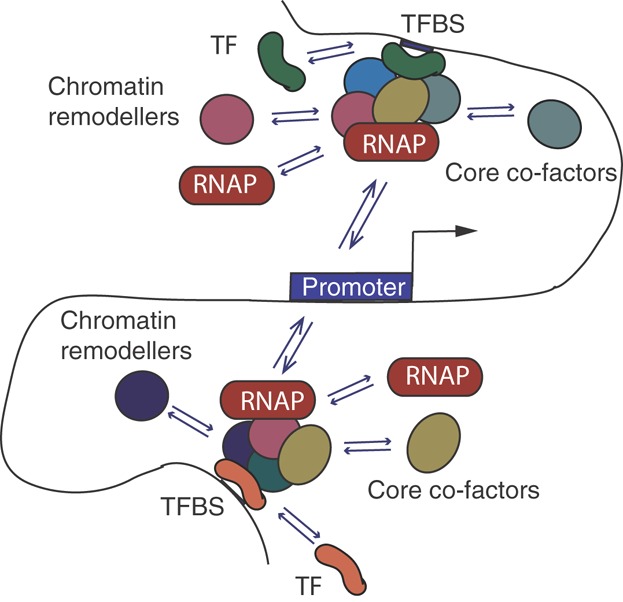
The ‘hit and run’ model of transcriptional activation. Under this model, transient interactions between transcription factors (TFs) and their binding sites on the DNA (TFBSs) promote the recruitment of chromatin remodellers, core transcriptional co-factors and the RNA polymerase (RNAP). These complexes, also transient in nature, stochastically ‘hit’ promoter regions, resulting in transcriptional initiation. The exact identity of both the TFs themselves and the co-factors they recruit need not be the same in each case, as emphasised by their different colours at the TFBSs ‘upstream’ and ‘downstream’ of the promoter. This model, based on evidence from time-course ChIP and FRAP 28–32, provides a mechanistic explanation of how multiple probabilistic events in gene regulation can lead to deterministic outcomes. TFBSs are depicted to localise some distance away from the promoter, but this model likely also applies to TFBSs located directly at the promoter region. For simplicity, RNAP and core co-factors are shown as freely distributed in the nuclear environment. There is however evidence to suggest that they are enriched at specific nuclear foci termed ‘transcription factories’ 24,25.

Understanding the mechanics of transcriptional activation is complicated by the startling diversity of core co-factors involved in this process. For example, the human genome contains four families of ATP-dependent chromatin remodelling complexes, at least four families of histone acetyltransferases, and a multitude of histone modifying enzymes such as methyltransferases and ubiquitin ligases that selectively modify specific histone amino acid residues 26. The early ‘deterministic’ models of transcriptional activation that postulated the existence of a well-orchestrated sequence of events involving ready-to-use core holoenzymes 27 made it difficult to accommodate this diversity of components. Indeed, deterministic models would presume the presence of a near-infinite number of highly specialised holoenzyme complexes, each of which is selectively required for the activation of specific subsets of genes under specific conditions. This deterministic view has however been challenged by studies that directly monitored the sequence of events underlying transcriptional activation in mammalian systems using techniques such as time-course immunoprecipitation 28 and fluorescent recovery after photobleaching (FRAP) 29,30. These analyses revealed that the well-defined deterministic stages of this process (such as chromatin remodelling, pre-initiation complex assembly, and transcriptional initiation) are likely to each comprise a series of transient ‘hit-and-run’ interactions of multiple proteins with each other and the DNA. The exact identity of these interactions and their order of action is flexible and to a degree stochastic 28–32 (Fig. 1).

One implication of this ‘probabilistic’ model of transcriptional initiation is that the scenarios of transcriptional activation are likely to be diverse and flexible even for a single gene and condition. Therefore, functionally TFs and co-factors may exhibit a partial redundancy even when their exact biochemical activities and ranges of interaction partners do not completely overlap 33–37. Further, this model suggests that transcriptional activation does not have to originate from a single TFBS or even a single regulatory module containing multiple TFBSs. Rather, multiple regions (containing one or more TFBSs) can each supply activating inputs to a gene's promoter 38,39. It is indeed frequently observed that a gene is regulated by multiple enhancers, each of which is independently capable of inducing a similar spatiotemporal pattern of expression in transgenic reporter assays. Such enhancers are sometimes referred to as ‘shadow enhancers’ (particularly if they were identified in addition to an earlier-characterised enhancer for the same gene) 38,40. Which of the ‘shadow enhancers’ associates with a target promoter at a given point of time may be down to random chance – as suggested, for example by the diversity of DNA looping interactions observed across single cells 41. Further, the fact that transcription likely occurs in discrete ‘bursts’ 42 makes it possible that even a seemingly continuous level of a gene's expression may in fact correspond to a series of ‘bursts’ initiated from multiple regulatory regions.

In conclusion, evidence reviewed above suggests that scenarios leading to transcriptional activation are likely to be flexible and to a degree stochastic, allowing for gene activation to be triggered from multiple regulatory regions, each containing one or more TFBSs. This model of probabilistic events combining into deterministic outcomes 43,44 is consistent with our current understanding of other biological processes such as DNA repair and lineage specification 45–47.

## The output of TF binding as a ‘dose of activation’

The flexibility of scenarios leading to transcriptional activation and the possibility of transcriptional initiation from multiple TFBSs suggest that there may not be a qualitative distinction between a ‘functional’ and a ‘spurious’ TFBS. Instead, each TFBS can be principally capable of contributing a ‘dose’ of activating signal to one or more promoters in its local chromosomal environment. Promoters, in turn, will respond to the total ‘dose’ transmitted to them by multiple TFBSs, including those located directly at promoter regions and those capable of coming into proximity with promoters through DNA looping interactions. This model does not imply that the contribution of each TFBS is useful in every case. Rather, it provides a framework for considering when and when not TFBS inputs are likely to be biologically meaningful. It also suggests that the total dose of activation may represent an evolutionary trait, as will be discussed later in this section.

### Doses large and small

TFBSs are known to have different binding affinities (or ‘strengths’) that are determined by factors such as the goodness-of-fit to the respective TFs' idealised sequence binding motif and the local chromatin accessibility 48,49. Considering a thermodynamic equilibrium between the TF-bound and unbound states, affinity can be seen as the probability that a TFBS is occupied by a TF at a given point of time. It is therefore reasonable to assume that higher ‘doses of activation’ would be generated by TFBSs that are embedded into accessible chromatin and whose sequences match the consensus motif, while poorly accessible TFBSs or those that have a poorer match to the sequence consensus would generate weaker ‘doses’. There is evidence suggesting that the probabilities of TF occupancy are non-zero for nearly every TFBS (or, in other words, that nearly all TFBSs are bound at least very weakly) 3,4,15,50, and it is clear that some of these binding events are weak enough to consider them negligible in nearly any situation. Yet, since the joint contribution of multiple ‘doses’ is likely to determine transcriptional outcomes, it may be difficult to infer functionality of a given TFBS in isolation from others. A more informed prediction could be obtained in considering the ‘dose of activation’ that a TFBS generates relative to the total ‘dose’ received by a gene of interest. This perspective is in line with the ‘thermodynamic’ models of gene regulation that combine multiple transcriptional regulatory events such as TF binding into a single probabilistic framework 51,52.

### ‘Dose of activation’ as an evolutionary trait

Verifying the ‘dose of activation’ model explicitly is challenging in most real-life cases, because of the large number of required perturbations. However, insights into this are offered by studies that compare TF binding and gene expression across multiple individuals of the same population 53–55. With individuals accumulating appreciable numbers of germline mutations in each generation (for humans, for example this number is estimated to be ∼74 56), each individual genome can be considered as a natural ‘perturbation’. Using this approach it was found, for example that the *fraction* of TFBSs in a gene's proximity that shows variable binding across individuals serves as a strong predictor of whether or not the target gene's expression itself is also variable 55. It can be argued that if TFBSs primarily segregated into ‘functional’ and ‘non-functional’, mutations at a subset of specific TFBSs, rather than the overall number of mutated TFBSs, would determine the effect on gene expression. Consistent with this, it has been recently demonstrated that the effects of TF knockdowns on their target genes' expression also correlate with the number of binding sites for a given TF in a gene's vicinity 57.

Evolutionary analyses looking at the conservation of TFBS sequences and binding events also provide support to the ‘dose of activation’ model. The assumption of the generally cumulative TFBS outputs implies that evolutionary selection would primarily act to preserve the total ‘dose of activation’ transmitted to a promoter, rather than the identity of each TFBS per se. Consistent with this, TFBSs often undergo a rapid turnover in evolution, disappearing and reappearing in proximity of their target genes 58–60. Although gene expression patterns are often evolutionarily conserved, regulatory regions that control them often have low base-to-base evolutionary conservation 6,61–63, and the divergence of TF binding events across species does not necessarily result in variation in their target gene expression 64.

An important corollary of these findings is that ‘base-by-base’ evolutionary conservation across species (which is a very useful proxy for functionality in the analyses of protein-coding regions) needs to be interpreted with caution in the case of regulatory sequence. For example highly conserved regulatory modules such as fly *even skipped* enhancers may appear neutral in such analyses 6. At the same time, the directly detectable sequence conservation of some other regulatory modules may in fact be a ‘side effect’ of an unrelated evolutionary process 65, such as the progressive loss of unconstrained sequences in between TFBSs 66.

### Do ‘doses’ add up at multi-TF enhancers?

The flexibility of sequence constraints at regulatory regions co-occupied by multiple TFs raises the possibility that even at these loci the output of each TF can be largely cumulative rather than synergistic. It should be noted however that this view is at odds with the classic ‘enhanceosome’ model whereby TFs are co-recruited as tightly organised complexes by means of precisely positioned consensus sequences for each TF, as has been extensively demonstrated for the β-interferon enhancer region 67. Yet, subsequent analyses on a broader range of regulatory modules have suggested that this model is unlikely to be universal 68,69. While the ‘enhanceosome’ may represent a type of enhancer organisation that is best suited for a rapid response to an environmental trigger such as viral infection, other regulatory regions seem to have a more flexible organisation. At these other regions, and particularly at developmental enhancers, different TFs may act seemingly independently as separate ‘symbols’ on a ‘billboard’ 68. Consistent with this, studies using thermodynamic modelling to predict the output of multi-TF enhancers have shown only a modest impact of activator-activator cooperativity 70,71. Although functional synergy in TF output (e.g. the exclusive ability of two or more TFs, but not any one of them, to recruit a specific co-factor) may exist at other regions, these results suggest that TF colocalisation at enhancer regions is not sufficient to assume cooperativity.

It is known however that many multi-TF regulatory regions do not contain recognisable sequence motifs for each of the TFs they recruit. This is the case, for example for the aforementioned HOT regions 12, many of which are known to act as early developmental enhancers 72. Partially, this phenomenon can be explained by the inevitable limitations of models used for predicting the TFs' DNA binding preferences 73,74. However, it is also possible that TF recruitment to these regions is facilitated or strengthened by protein-protein interactions 69,75–77, as in the ‘TF collective’ model that we have recently proposed 69,78. Consistent with this, knocking out one TF recruited to multi-TF regulatory regions was shown to destabilise the binding of other TFs 79. In addition, it is known that many TFs are unable to bind DNA through repressed chromatin and their recruitment is dependent on the so-called ‘pioneer TFs’ that possess this ability 80.

Scenarios whereby TFs depend on each other to secure their recruitment to DNA may create a paradoxical situation, whereby the clearly observable biochemical cooperativity between TFs does not preclude the generally independent functional outputs of each factor. For example, TF recruitment to HOT regions is likely to involve some synergy between TFs, as these regions often do not feature the full set of sequence motifs for each TF found at them 12. However, the transcriptional outputs of HOT regions are still generally proportional to the number of co-bound TFs 81, suggesting the generally independent functional outputs of each bound TF. It is possible that similar mechanisms underlie some of the synergistic effects reported for a number of other multi-TF regulatory modules 82,83.

In conclusion, considerations from transcriptional mechanics, as well as evidence from the global analyses of TF binding across individuals and species, point towards a largely cumulative view of TFBS functional outputs. Some deviations from the simple sum of individual TFBS activities can be expected, particularly at regulatory regions recruiting multiple TFs. However, the ‘dose of activation’ model is helpful for interpreting TFBS function and particularly the effects of TFBS aberrations that may otherwise seem unexpected, as will be discussed in the next section.

## TFBS genetic redundancy: Why and so what?

**Figure 2:**
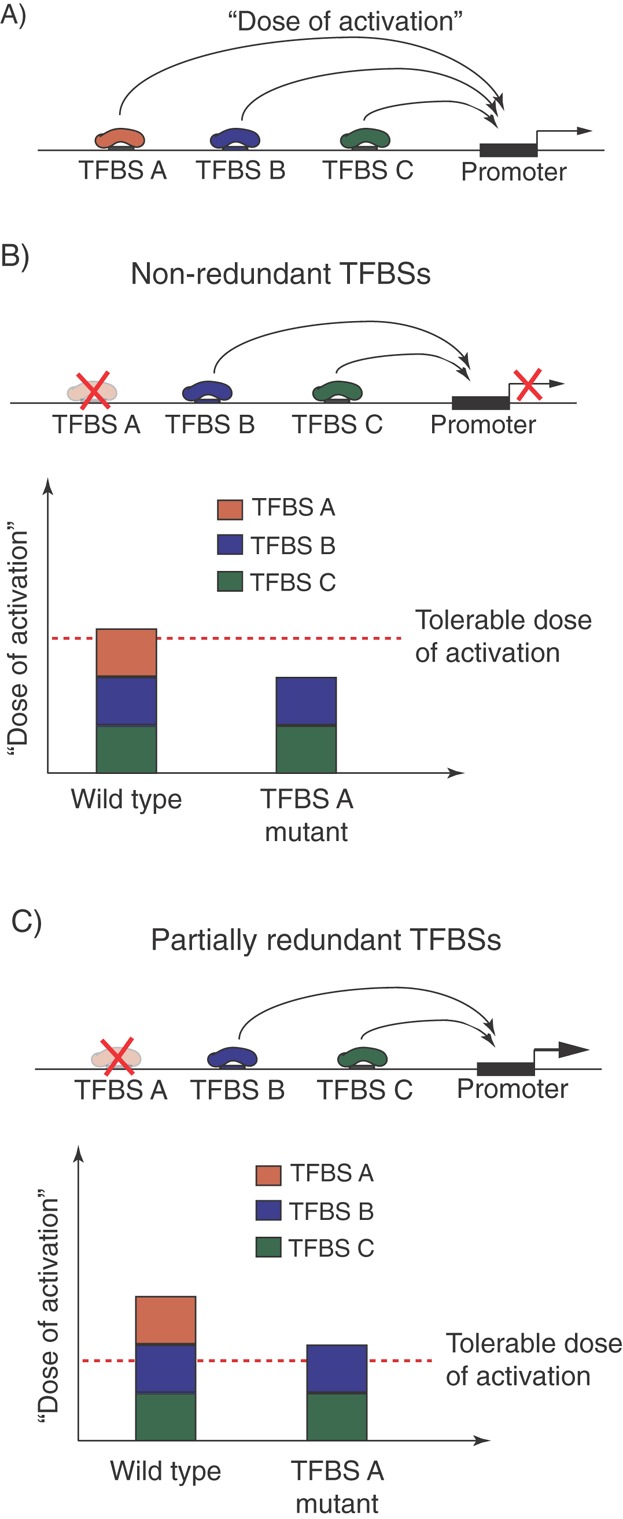
The ‘dose of activation’ view of TFBS action and the emergence of redundancy. A: The probabilistic model of transcriptional initiation involving transient ‘hit-and-run’ interactions (Fig. 1) suggests that TF-binding events at multiple TFBSs provide generally cumulative inputs (‘doses of activation’) to their target promoters. B: When the total input from a group of TFBSs narrowly reaches the minimum tolerable ‘dose’, mutations at individual TFBSs will be poorly tolerated. C: If, on the other hand, the total inputs exceed this dose, the system becomes partially redundant and mutations at individual TFBSs may not cause significant changes in gene expression.

Let us consider a hypothetical gene promoter that receives inputs from several TFBSs (Fig. 2A). If the total ‘dose of activation’ jointly transmitted to it narrowly reaches the biologically admissible threshold, each TFBS is likely to be deemed ‘functional’ in perturbation analyses, as its deletion will result in a ‘dose’ insufficient for achieving the minimally functional level of a gene's expression (Fig. 2B). However, if their total contribution exceeds this threshold, at least under some conditions, the system becomes partially redundant, rendering the transcriptional output relatively insensitive to perturbations at individual TFBSs (Fig. 2C). Here, I will consider the likely causes and consequences of such redundancy.

### TFBS redundancy is likely widespread

Several lines of evidence suggest that redundancy across TFBSs is likely to be widespread. For example although TFBS mutations underlie many genotype-disease associations 84,85, they are known to be responsible for only a handful of Mendelian disorders 86. Consistent with this, the majority of individual TFBS polymorphisms identified at the population level associate with little to no deviation in gene expression 54,55. However, as discussed above, the impact of these polymorphisms on gene expression increases proportionately with the number of affected TFBSs 55. The tolerance of enhancer outputs to single-TFBS mutations has also been demonstrated in targeted perturbation experiments for some regions 9,87. Collectively, this evidence suggests that in contrast to many protein-coding mutations, TFBS mutations are often ‘buffered’ by the regulatory network, rendering many, if not most individual TFBS contributions partially redundant.

### An adaptive trait or a side effect?

Although initially unexpected from the classic evolutionary theory, the broader phenomenon of the so-called genetic redundancy has been extensively characterised at the level of homologous genes and network modules in various systems 88–90. The well-known examples of genetic redundancy include genes generated by gene duplication, many of which will subsequently pseudogenise or diverge in evolution. However, the evolutionary retention of duplicate genes is also relatively common 91. Perhaps even more common is the functional redundancy at the level of regulatory and metabolic networks 2,90,92,93. For instance, up to ∼45% of metabolic reactions in *Escherichia coli* and yeast can be individually removed without significantly affecting the production of any biomass component under multiple nutritional conditions 92. Likewise, redundancy is often observed between the inputs of multiple signalling pathways, presenting a significant obstacle in using individual signalling pathway inhibitors in the treatment of cancer and inflammatory conditions 94,95.

While it is generally accepted that partial redundancy is an intrinsic property of complex networks 96,97, it may also be an adaptive trait, serving, for example to downplay the effects of noise on transcriptional outputs 98. In line with this, although the emergence of redundant groups of TFBSs regulating the same gene can theoretically offer a selective advantage, it can also represent a side effect of other factors. For example it has been predicted that multiple weaker sites (a scenario that predisposes to partial redundancy) may be easier to evolve compared to fewer strong ones 65. At the same time, weaker sites can also be specifically advantageous at genes that require finely tuned regulation of expression levels 99.

### The cryptic consequences of TFBS redundancy

Extending the concept of genetic redundancy to TFBSs has several important implications. First, it suggests that the degree of redundancy may be a key factor in determining a TFBS's observed functional constraint, even when detected by more precise proxies than cross-species conservation, such as within-species variability. In particular, it has been shown that TFBSs that are ‘backed-up’ by other TFBSs, located either in their direct proximity or at additional regulatory modules regulating the same gene, are less constrained than their less redundant counterparts 9,54,55,100. It is therefore possible that genes that receive inputs from multiple, possibly weak TFBSs are regulated more robustly than those controlled by a small number of stronger sites. This may have a significant impact on the phenotypic consequences of TFBS mutations, such as their chances to lead to disease phenotypes.

**Figure 3:**
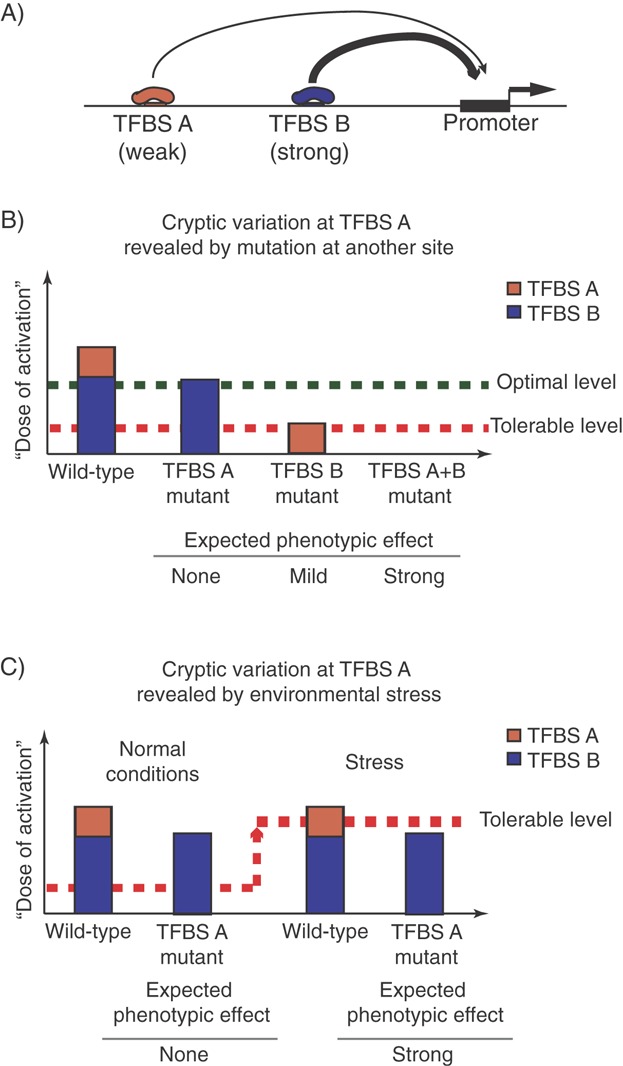
Genetic and environmental perturbations may uncover the cryptic impact of redundant transcription factor binding sites. A: Example of a hypothetical promoter receiving activating inputs from a low-affinity TFBS A (‘weak’, orange) and a high-affinity TFBS B (‘strong’, blue). B: Assuming that the strong TFBS B generates a sufficient ‘dose of activation’ to achieve the optimal level of gene expression. Under these conditions, input from the weak TFBS A is redundant and deleterious mutations at TFBS A are expected to produce little to no phenotypic effects. However, TFBS A may be able to at least partially buffer the effects of TFBS B mutation, ensuring that the total ‘dose of activation’ does not fall below the minimally tolerable level. C: The contribution of a weak TFBS A to the transcriptional output may also be revealed by changes in the environment (‘stress’) that result in an increase in the minimally tolerable ‘dose of activation’. In this scenario, TFBS A deletion may not have a phenotype under normal conditions, but show strong phenotypic effects under stress conditions.

A second, potentially counterintuitive consequence of genetic redundancy is that groups of TFBSs may be in epistatic relationships with each other that are not observable under normal conditions. While classically described for redundant protein-coding genes 101,102, this phenomenon likely also applies to regulatory sequences. In particular, variation at a TFBS that seems unconstrained (or ‘spurious’) in healthy individuals may turn out to determine whether mutations at other, perhaps ‘stronger’ TFBSs will lead to disease onset (Fig. 3A). One real-life example of this can be seen in the regulatory logic of the homeobox gene *cog-1* in *Caenorhabditis elegans*. This gene is controlled by a zinc-finger TF (CHE1) that is recruited to two TFBSs in the *cog-1* upstream region. It has been shown that the deletion of the weaker ‘distal’ TFBS does not affect the levels of *cog-1* expression, at least in an in vitro reporter assay. However, when the stronger ‘proximal’ CHE1 TFBS is deleted, the ‘distal’ TFBS is able to maintain 50% of the normal *cog-1* levels. The deletion of both ‘proximal’ and ‘distal’ TFBSs abolishes *cog-1* expression altogether 103.

Redundancy between TFBSs may also be broken because of suboptimal environmental conditions that increase the ‘dose of activation’ required to achieve functional levels of gene expression (Fig. 3B). These ‘stress’ conditions may include changes in the extra-organismal environment, such as availability of specific nutrients 104 or varying temperature 105. For instance, it has been shown that removing two enhancer regions of *Drosophila svb* gene produces no phenotype under normal conditions, but leads to embryonic defects under abnormally low and high temperatures 106.

No less importantly, the impact of apparently redundant TFBSs may also be unmasked by homeostatic changes within the organism itself. For example in nematodes ablation of gonadal signalling may or may not lead to abnormal vulva development depending on a TFBS polymorphism that is otherwise phenotypically neutral 107. This buffering of cis-regulatory variation by inputs from signalling pathways is also potentially relevant in ageing, where both the ability of the cells to receive extracellular signals and the signalling potency of the cellular microenvironment are diminished 108,109. Consistent with this expectation, a number of expression quantitative trait loci (eQTLs) associated with complex diseases have recently been found to show selective effects during ageing 110. Finally, TFBS variability can be revealed by targeted changes in cell homeostasis, such as by treatment with drugs that modulate specific signalling pathways 111–113. Since fitness for both ageing and medical treatment is not subject to a strong evolutionary pressure, it is conceivable that fitness advantages in these situations will be provided by elements that are genuinely unconstrained in evolution, such as low-affinity TFBSs.

The phenomenon of ‘cryptic variability’ that is only uncovered by genetic or environmental perturbations has been described in other systems and is thought to be an important modifier of phenotypic response 114. Consistent with this, epistatic relationships between multiple regulatory elements pose a major challenge in the interpretation of genome-wide association studies 115,116. Therefore, the question of whether an apparently unconstrained TFBS is ‘non-functional’ or forms part of a partially redundant regulatory unit is not esoteric. Rather, it has clear implications for the interpretation of non-coding mutations. While it may be tempting to adopt the conservative approach of ‘non-functional until proven otherwise’ to weak or poorly evolutionary constrained TF-binding events, they may turn out to be pivotal for determining whether or not the system will cope with environmental or genetic stress.

It would be useful to predict the degree of genetic redundancy between TFBSs from parameters other than their variability within or across species. Doing so would help reveal cryptic epistatic relationships, as well as potentially uncover TFBSs that are genuinely ‘spurious’. A crude way to estimate the redundancy of a given TFBS with respect to a given gene's regulation would be to estimate how many other TFBSs form (or can form) looping interactions with its promoter in a given cell type and condition. Advances of functional genomics and particularly the high-throughput modifications of the chromosome conformation capture technique 117,118 are now making it possible to directly address this question in some situations. However, further studies are needed to understand the ‘logic’ of long-range interactions, and massively parallel analyses such as TRIP 119 are paving the way in this direction.

In conclusion, TFBSs are often at least partially redundant, which can be a side effect of network complexity, but potentially also an adaptive trait serving to increase system robustness. As a result, TFBS mutations may produce little to no phenotype not only when they genuinely have no impact on target promoters, but also because their inputs are efficiently ‘buffered’ by other TFBSs. Since the buffering capacity depends on other TFBSs being intact as well as on the environmental conditions, a significant fraction of TFBS variation may be phenotypically ‘cryptic’. This may have implications for interpreting TFBS function, particularly under suboptimal conditions such as old age and disease.

## Conclusions

Collective evidence from the molecular mechanics of transcriptional regulation, functional genomics, evo-devo and population genetics discussed in this essay suggest that rather than considering multiple TF binding events in the proximity of a gene as either ‘functional’ or ‘spurious’, it is perhaps more appropriate to view them as jointly contributing incremental ‘doses’ of transcriptional activation to the total pool that can differ in potency from high to negligibly low. Redundancy emerges in this system when the total ‘dose’ exceeds the ‘threshold of activation’ required to generate an adequate level of a gene's expression. Because of such redundancy, some TFBSs appear near neutral, both evolutionary and functionally, under normal conditions. These same sites however may play pivotal roles when the system is pushed away from the optimum by either genetic or environmental abnormalities. With the majority of disease-associated SNPs mapping to non-coding, potentially regulatory regions 100, it is important to gain a better understanding of how multiple TF binding events integrate to regulate transcription in time and space. Accounting for the variable levels of TFBS redundancy under different conditions may improve our ability to interpret regulatory mutations.

## Abbreviations

ChIP: pchromatin immunoprecipitation
eQTL: expression quantitative trait locus
HOT: high occupancy TF
TF: factor
TFBS: transcription factor binding site

## References

[b1] Kadonaga JT (2004). Regulation of RNA polymerase II transcription by sequence-specific DNA binding factors. Cell.

[b2] Wray GA (2007). The evolutionary significance of cis-regulatory mutations. Nat Rev Genet.

[b3] Li X-Y, MacArthur S, Bourgon R, Nix D (2008). Transcription factors bind thousands of active and inactive regions in the Drosophila blastoderm. PLoS Biol.

[b4] Biggin MD (2011). Animal transcription networks as highly connected, quantitative continua. Dev Cell.

[b5] Fisher WW, Li JJ, Hammonds AS, Brown JB (2012). DNA regions bound at low occupancy by transcription factors do not drive patterned reporter gene expression in Drosophila. Proc Natl Acad Sci USA.

[b6] Hare EE, Peterson BK, Iyer VN, Meier R (2008). Sepsid even-skipped enhancers are functionally conserved in Drosophila despite lack of sequence conservation. PLoS Genet.

[b7] Meireles-Filho AC, Stark A (2009). Comparative genomics of gene regulation – conservation and divergence of cis-regulatory information. Curr Opin Genet Dev.

[b8] Maurano MT, Wang H, Kutyavin T, Stamatoyannopoulos JA (2012). Widespread site-dependent buffering of human regulatory polymorphism. PLoS Genet.

[b9] Spivakov M, Akhtar J, Kheradpour P, Beal K (2012). Analysis of variation at transcription factor binding sites in Drosophila and humans. Genome Biol.

[b10] Moorman C, Sun LV, Wang J, de Wit E (2006). Hotspots of transcription factor colocalization in the genome of *Drosophila melanogaster*. Proc Natl Acad Sci USA.

[b11] Gerstein MB, Lu ZJ, Van Nostrand EL, Cheng C (2010). Integrative analysis of the *Caenorhabditis elegans* genome by the modENCODE project. Science.

[b12] Roy S, Ernst J, Kharchenko PV, The modENCODE Consortium (2010). Identification of functional elements and regulatory circuits by Drosophila modENCODE. Science.

[b13] The ENCODE Project Consortium (2012). An integrated encyclopedia of DNA elements in the human genome. Nature.

[b14] Cooper GM, Brown CD (2008). Qualifying the relationship between sequence conservation and molecular function. Genome Res.

[b15] MacArthur S, Li X-Y, Li J, Brown JB (2009). Developmental roles of 21 Drosophila transcription factors are determined by quantitative differences in binding to an overlapping set of thousands of genomic regions. Genome Biol.

[b16] Perissi V, Jepsen K, Glass CK, Rosenfeld MG (2010). Deconstructing repression: evolving models of co-repressor action. Nat Rev Genet.

[b17] Merkenschlager M, Odom DT (2013). CTCF and cohesin: linking gene regulatory elements with their targets. Cell.

[b18] van Oevelen C, Kallin EM, Graf T (2013). Transcription factor-induced enhancer modulations during cell fate conversions. Curr Opin Genet Dev.

[b19] Taatjes DJ, Marr MT, Tjian R (2004). Opinion: regulatory diversity among metazoan co-activator complexes. Nat Rev Mol Cell Biol.

[b20] Malik S, Roeder RG (2010). The metazoan mediator co-activator complex as an integrative hub for transcriptional regulation. Nat Rev Genet.

[b21] Adelman K, Lis JT (2012). Promoter-proximal pausing of RNA polymerase II: emerging roles in metazoans. Nat Rev Genet.

[b22] Symmons O, Spitz F (2013). From remote enhancers to gene regulation: charting the genome's regulatory landscapes. Philos Trans R Soc Lond B Biol Sci.

[b23] Wendt KS, Grosveld FG (2014). Transcription in the context of the 3D nucleus. Curr Opin Genet Dev.

[b24] Osborne CS, Chakalova L, Brown KE, Carter D (2004). Active genes dynamically colocalize to shared sites of ongoing transcription. Nat Genet.

[b25] Ghamari A, van de Corput MPC, Thongjuea S, van Cappellen WA (2013). In vivo live imaging of RNA polymerase II transcription factories in primary cells. Genes Dev.

[b26] Clapier CR, Cairns BR (2009). The biology of chromatin remodeling complexes. Annu Rev Biochem.

[b27] Parvin JD, Young RA (1998). Regulatory targets in the RNA polymerase II holoenzyme. Curr Opin Genet Dev.

[b28] Métivier R, Penot G, Hübner MR, Reid G (2003). Estrogen receptor-α directs ordered, cyclical, and combinatorial recruitment of cofactors on a natural target promoter. Cell.

[b29] Kimura H, Sugaya K, Cook PR (2002). The transcription cycle of RNA polymerase II in living cells. J Cell Biol.

[b30] Dundr M, Hoffmann-Rohrer U, Hu Q, Grummt I (2002). A kinetic framework for a mammalian RNA polymerase in vivo. Science.

[b31] Becker M, Baumann C, John S, Walker DA (2002). Dynamic behavior of transcription factors on a natural promoter in living cells. EMBO Rep.

[b32] Vermeulen W, Houtsmuller AB (2002). The transcription cycle in vivo: a blind watchmaker at work. Mol Cell.

[b33] Santisteban MS, Kalashnikova T, Smith MM (2000). Histone H2A.Z. regulates transcription and is partially redundant with nucleosome remodeling complexes. Cell.

[b34] Martin AM, Pouchnik DJ, Walker JL, Wyrick JJ (2004). Redundant roles for histone H3 N-terminal lysine residues in subtelomeric gene repression in *Saccharomyces cerevisiae*. Genetics.

[b35] Barbaric S, Luckenbach T, Schmid A, Blaschke D (2007). Redundancy of chromatin remodeling pathways for the induction of the yeast PHO5 promoter in vivo. J Biol Chem.

[b36] Bezhani S, Winter C, Hershman S, Wagner JD (2007). Unique, shared, and redundant roles for the Arabidopsis SWI/SNF chromatin remodeling ATPases BRAHMA and SPLAYED. Plant Cell.

[b37] Kelly RDW, Cowley SM (2013). The physiological roles of histone deacetylase (HDAC) 1 and 2: complex co-stars with multiple leading parts. Biochem Soc Trans.

[b38] Barolo S (2011). Shadow enhancers: frequently asked questions about distributed cis-regulatory information and enhancer redundancy. Bioessays.

[b39] Samee MAH, Sinha S (2014). Quantitative modeling of a gene's expression from its intergenic sequence. PLoS Comput Biol.

[b40] Levine M (2010). Transcriptional enhancers in animal development and evolution. Curr Biol.

[b41] Nagano T, Lubling Y, Stevens TJ, Schoenfelder S (2013). Single-cell Hi-C reveals cell-to-cell variability in chromosome structure. Nature.

[b42] Raj A, van Oudenaarden A (2008). Nature, nurture, or chance: stochastic gene expression and its consequences. Cell.

[b43] Verzijlbergen KF, Faber AW, Stulemeijer IJ, van Leeuwen F (2009). Multiple histone modifications in euchromatin promote heterochromatin formation by redundant mechanisms in *Saccharomyces cerevisiae*. BMC Mol Biol.

[b44] Coulon A, Chow CC, Singer RH, Larson DR (2013). Eukaryotic transcriptional dynamics: from single molecules to cell populations. Nat Rev Genet.

[b45] O'Brien PJ (2006). Catalytic promiscuity and the divergent evolution of DNA repair enzymes. Chem Rev.

[b46] Eldar A, Elowitz MB (2010). Functional roles for noise in genetic circuits. Nature.

[b47] Teles J, Pina C, Edén P, Ohlsson M (2013). Transcriptional regulation of lineage commitment – a stochastic model of cell fate decisions. PLoS Comput Biol.

[b48] Bulyk ML (2003). Computational prediction of transcription-factor binding site locations. Genome Biol.

[b49] Neph S, Vierstra J, Stergachis AB, Reynolds AP (2012). An expansive human regulatory lexicon encoded in transcription factor footprints. Nature.

[b50] Elf J, Li G-W, Xie XS (2007). Probing transcription factor dynamics at the single-molecule level in a living cell. Science.

[b51] Bintu L, Buchler NE, Garcia HG, Gerland U (2005). Transcriptional regulation by the numbers: models. Curr Opin Genet Dev.

[b52] Roider HG, Kanhere A, Manke T, Vingron M (2007). Predicting transcription factor affinities to DNA from a biophysical model. Bioinformatics.

[b53] Kasowski M, Grubert F, Heffelfinger C, Hariharan M (2010). Variation in transcription factor binding among humans. Science.

[b54] Kilpinen H, Waszak SM, Gschwind AR, Raghav SK (2013). Coordinated effects of sequence variation on DNA binding, chromatin structure, and transcription. Science.

[b55] Kasowski M, Kyriazopoulou-Panagiotopoulou S, Grubert F, Zaugg JB (2013). Extensive variation in chromatin states across humans. Science.

[b56] Conrad DF, Keebler JEM, DePristo MA, Lindsay SJ (2011). Variation in genome-wide mutation rates within and between human families. Nat Genet.

[b57] Cusanovich DA, Pavlovic B, Pritchard JK, Gilad Y (2014). The functional consequences of variation in transcription factor binding. PLoS Genet.

[b58] Moses AM, Pollard DA, Nix DA, Iyer VN (2006). Large-scale turnover of functional transcription factor binding sites in Drosophila. PLoS Comput Biol.

[b59] Doniger SW, Fay JC (2007). Frequent gain and loss of functional transcription factor binding sites. PLoS Comput Biol.

[b60] Kim J, He X, Sinha S (2009). Evolution of regulatory sequences in 12 Drosophila species. PLoS Genet.

[b61] Dowell RD (2010). Transcription factor binding variation in the evolution of gene regulation. Trends Genet.

[b62] Schmidt D, Wilson MD, Ballester B, Schwalie PC (2010). Five-vertebrate ChIP-seq reveals the evolutionary dynamics of transcription factor binding. Science.

[b63] Habib N, Wapinski I, Margalit H, Regev A (2012). A functional selection model explains evolutionary robustness despite plasticity in regulatory networks. Mol Syst Biol.

[b64] Paris M, Kaplan T, Li X-Y, Villalta JE (2013). Extensive divergence of transcription factor binding in Drosophila embryos with highly conserved gene expression. PLoS Genet.

[b65] He X, Duque TSPC, Sinha S (2012). Evolutionary origins of transcription factor binding site clusters. Mol Biol Evol.

[b66] Lusk RW, Eisen MB (2010). Evolutionary mirages: selection on binding site composition creates the illusion of conserved grammars in Drosophila enhancers. PLoS Genet.

[b67] Thanos D, Maniatis T (1995). Virus induction of human IFNβ gene expression requires the assembly of an enhanceosome. Cell.

[b68] Arnosti DN, Kulkarni MM (2005). Transcriptional enhancers: intelligent enhanceosomes or flexible billboards. J Cell Biochem.

[b69] Spitz F, Furlong EEM (2012). Transcription factors: from enhancer binding to developmental control. Nat Rev Genet.

[b70] Dresch JM, Liu X, Arnosti DN, Ay A (2010). Thermodynamic modeling of transcription: sensitivity analysis differentiates biological mechanism from mathematical model-induced effects. BMC Syst Biol.

[b71] He X, Samee MAH, Blatti C, Sinha S (2010). Thermodynamics-based models of transcriptional regulation by enhancers: the roles of synergistic activation, cooperative binding and short-range repression. PLoS Comput Biol.

[b72] Kvon EZ, Stampfel G, Yáñez-Cuna JO, Dickson BJ (2012). HOT regions function as patterned developmental enhancers and have a distinct cis-regulatory signature. Genes Dev.

[b73] Zhou Q, Liu JS (2004). Modeling within-motif dependence for transcription factor binding site predictions. Bioinformatics.

[b74] Narlikar L, Mehta N, Galande S, Arjunwadkar M (2013). One size does not fit all: on how Markov model order dictates performance of genomic sequence analyses. Nucleic Acids Res.

[b75] Gordân R, Hartemink AJ, Bulyk ML (2009). Distinguishing direct versus indirect transcription factor–DNA interactions. Genome Res.

[b76] Nagulapalli S, Atchison ML (1998). Transcription factor Pip can enhance DNA binding by E47, leading to transcriptional synergy involving multiple protein domains. Mol Cell Biol.

[b77] Omari ElK, Hoosdally SJ, Tuladhar K, Karia D (2013). Structural basis for LMO2-driven recruitment of the SCL:E47bHLH heterodimer to hematopoietic-specific transcriptional targets. Cell Rep.

[b78] Junion G, Spivakov M, Girardot C, Braun M (2012). A transcription factor collective defines cardiac cell fate and reflects lineage history. Cell.

[b79] Stefflova K, Thybert D, Wilson MD, Streeter I (2013). Cooperativity and rapid evolution of cobound transcription factors in closely related mammals. Cell.

[b80] Zaret KS, Carroll JS (2011). Pioneer transcription factors: establishing competence for gene expression. Genes Dev.

[b81] Foley JW, Sidow A (2013). Transcription-factor occupancy at HOT regions quantitatively predicts RNA polymerase recruitment in five human cell lines. BMC Genomics.

[b82] Swanson CI, Evans NC, Barolo S (2010). Structural rules and complex regulatory circuitry constrain expression of a Notch- and EGFR-regulated eye enhancer. Dev Cell.

[b83] Erceg J, Saunders TE, Girardot C, Devos DP (2014). Subtle changes in motif positioning cause tissue-specific effects on robustness of an enhancer's activity. PLoS Genet.

[b84] Manolio TA (2013). Bringing genome-wide association findings into clinical use. Nat Rev Genet.

[b85] Karczewski KJ, Dudley JT, Kukurba KR, Chen R (2013). Systematic functional regulatory assessment of disease-associated variants. Proc Natl Acad Sci USA.

[b86] Chakravarti A (2011). Genomic contributions to Mendelian disease. Genome Res.

[b87] Drewell RA (2011). Transcription factor binding site redundancy in embryonic enhancers of the Drosophila bithorax complex. G3 (Bethesda).

[b88] Thomas JH (1993). Thinking about genetic redundancy. Trends Genet.

[b89] Nowak MA, Boerlijst MC, Cooke J, Smith JM (1997). Evolution of genetic redundancy. Nature.

[b90] Zhang J (2012). Genetic redundancies and their evolutionary maintenance. Adv Exp Med Biol.

[b91] Vavouri T, Semple JI, Lehner B (2008). Widespread conservation of genetic redundancy during a billion years of eukaryotic evolution. Trends Genet.

[b92] Wang Z, Zhang J (2009). Abundant indispensable redundancies in cellular metabolic networks. Genome Biol Evol.

[b93] Paixão T, Azevedo RBR (2010). Redundancy and the evolution of cis-regulatory element multiplicity. PLoS Comput Biol.

[b94] Logue JS, Morrison DK (2012). Complexity in the signaling network: insights from the use of targeted inhibitors in cancer therapy. Genes Dev.

[b95] van Wageningen S, Kemmeren P, Lijnzaad P, Margaritis T (2010). Functional overlap and regulatory links shape genetic interactions between signaling pathways. Cell.

[b96] Hermisson J, Wagner GP, Jen E (2005). Evolution of phenotypic robustness. Robust Design: A Repertoire from Biology, Ecology, and Engineering.

[b97] MacArthur BD, Sánchez-García RJ, Anderson JW (2008). Symmetry in complex networks. Discrete Appl Math.

[b98] Kafri R, Springer M, Pilpel Y (2009). Genetic redundancy: new tricks for old genes. Trends Immunol.

[b99] Ramos AI, Barolo S (2013). Low-affinity transcription factor binding sites shape morphogen responses and enhancer evolution. Phil Trans R Soc B.

[b100] Maurano MT, Humbert R, Rynes E, Thurman RE (2012). Systematic localization of common disease-associated variation in regulatory DNA. Science.

[b101] Musso G, Costanzo M, Huangfu M, Smith AM (2008). The extensive and condition-dependent nature of epistasis among whole-genome duplicates in yeast. Genome Res.

[b102] Putty K, Marcus SA, Mittl PRE, Bogadi LE (2013). Robustness of *Helicobacter pylori* infection conferred by context-variable redundancy among cysteine-rich paralogs. PLoS One.

[b103] O'Meara MM, Bigelow H, Flibotte S, Etchberger JF (2009). Cis-regulatory mutations in the *Caenorhabditis elegans* homeobox gene locus cog-1 affect neuronal development. Genetics.

[b104] Bhatia A, Yadav A, Gagneur J, Zhu C (2014). Yeast growth plasticity is regulated by environment specific multi-QTL interactions. G3 (Bethesda).

[b105] de Nadal E, Ammerer G, Posas F (2011). Controlling gene expression in response to stress. Nat Rev Genet.

[b106] Frankel N, Davis GK, Vargas D, Wang S (2010). Phenotypic robustness conferred by apparently redundant transcriptional enhancers. Nature.

[b107] Kienle S, Sommer RJ (2013). Cryptic variation in vulva development by cis-regulatory evolution of a HAIRY-binding site. Nat Commun.

[b108] Strehler BL, Freeman MR (1980). Randomness, redundancy and repair: roles and relevance to biological aging. Mech Ageing Dev.

[b109] Han J-DJ (2012). An aging program at the systems level. Birth Defects Res C Embryo Today.

[b110] Yao C, Joehanes R, Johnson AD, Huan T (2014). Sex- and age-interacting eQTLs in human complex diseases. Hum Mol Genet.

[b111] Choy E, Yelensky R, Bonakdar S, Plenge RM (2008). Genetic analysis of human traits in vitro: drug response and gene expression in lymphoblastoid cell lines. PLoS Genet.

[b112] Maranville JC, Luca F, Richards AL, Wen X (2011). Interactions between glucocorticoid treatment and cis-regulatory polymorphisms contribute to cellular response phenotypes. PLoS Genet.

[b113] Gaffney DJ (2013). Global properties and functional complexity of human gene regulatory variation. PLoS Genet.

[b114] Gibson G, Dworkin I (2004). Uncovering cryptic genetic variation. Nat Rev Genet.

[b115] Moore JH, Williams SM (2009). Epistasis and its implications for personal genetics. Am J Hum Genet.

[b116] McKinney BA, Pajewski NM (2012). Six degrees of epistasis: statistical network models for GWAS. Front Genet.

[b117] Dekker J, Marti-Renom MA, Mirny LA (2013). Exploring the three-dimensional organization of genomes: interpreting chromatin interaction data. Nat Rev Genet.

[b118] Hughes JR, Roberts N, McGowan S, Hay D (2014). Analysis of hundreds of cis-regulatory landscapes at high resolution in a single, high-throughput experiment. Nat Genet.

[b119] Akhtar W, de Jong J, Pindyurin AV, Pagie L (2013). Chromatin position effects assayed by thousands of reporters integrated in parallel. Cell.

